# Supplementary material for: Beyond Quiescent and Active: Intermediate Microglial Transcriptomic States in a Mouse Model of Down Syndrome
Source: Int J Mol Sci. 2024 Mar 14;25(6):3289. doi: 10.3390/ijms25063289 (PMC10970051; doi:10.3390/ijms25063289)
Supplement: Supplementary file 1 [file ijms-25-03289-s001.zip › Supplementary Material_Microglia.pdf]

Supplementary Materials

Supplementary Figures

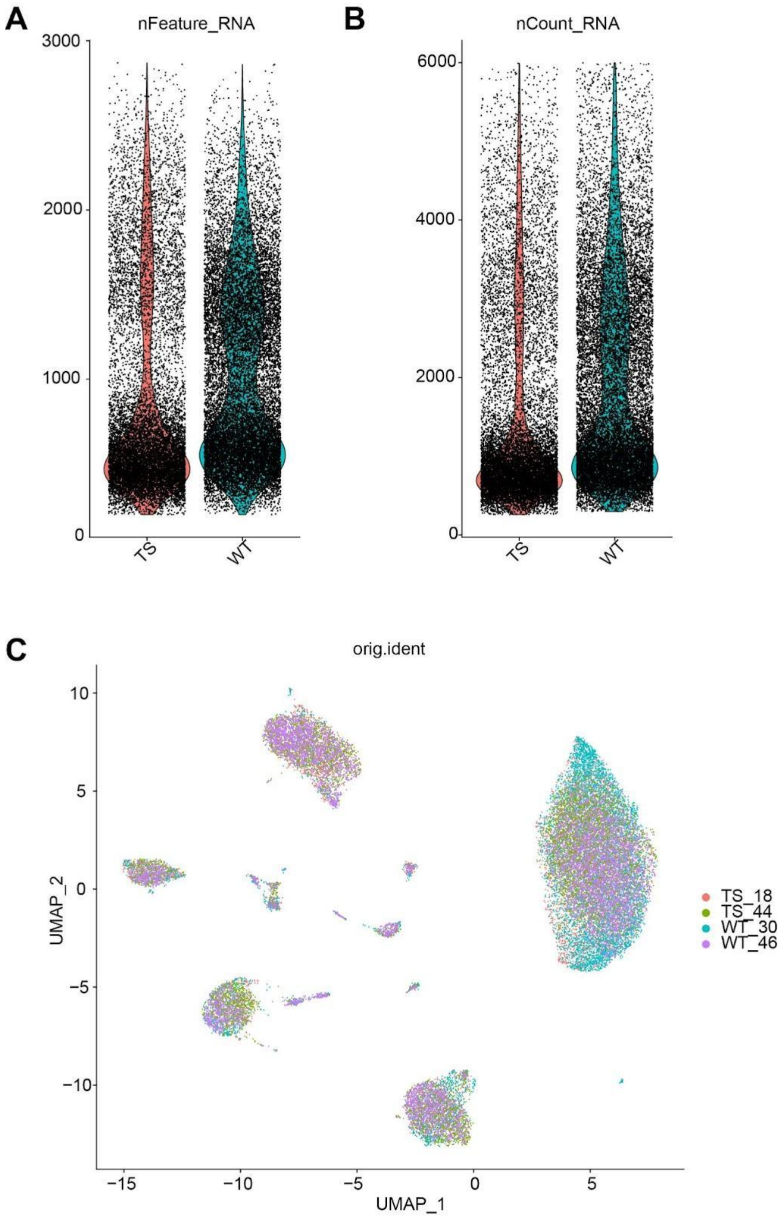

Supplementary Figure S1. (A) Violin plot for the number of unique genes detected per cell in each sample. **(B)** Violin plot for the number of unique transcripts detected per cell in each sample (WT = 2, Ts65Dn = 2). **(C)** UMAP representation of the nuclei transcriptome representing the sample batch effects on cell clusters. All the clusters are composed of a mixture of cells that originated from each of the four samples. Cells are colored by sample (WT_30, WT_46, TS_18 and TS 44).

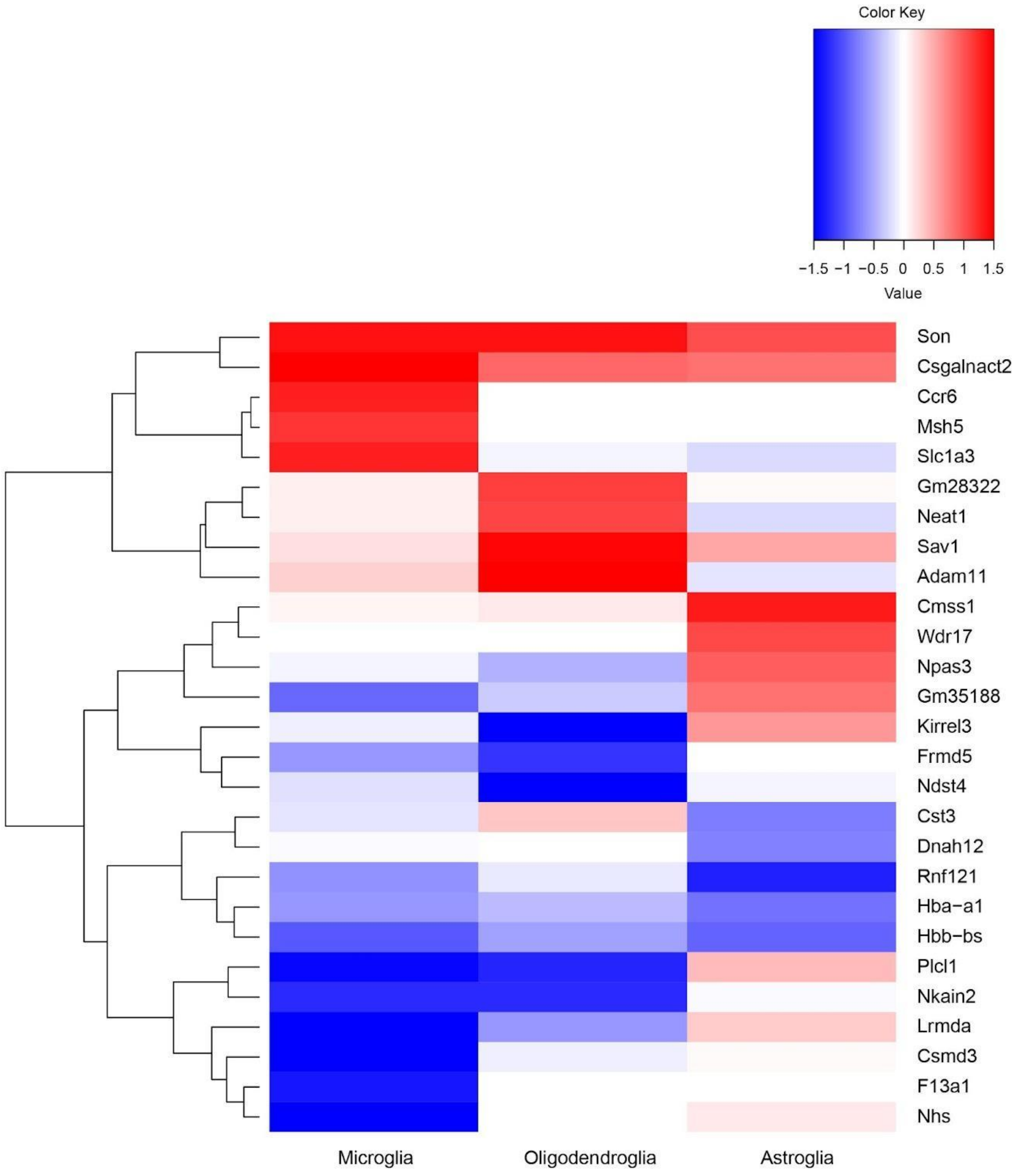

Supplementary Figure S2. Heatmap showing log2 FC values for the top DEGs (rows) in each glial type (columns).

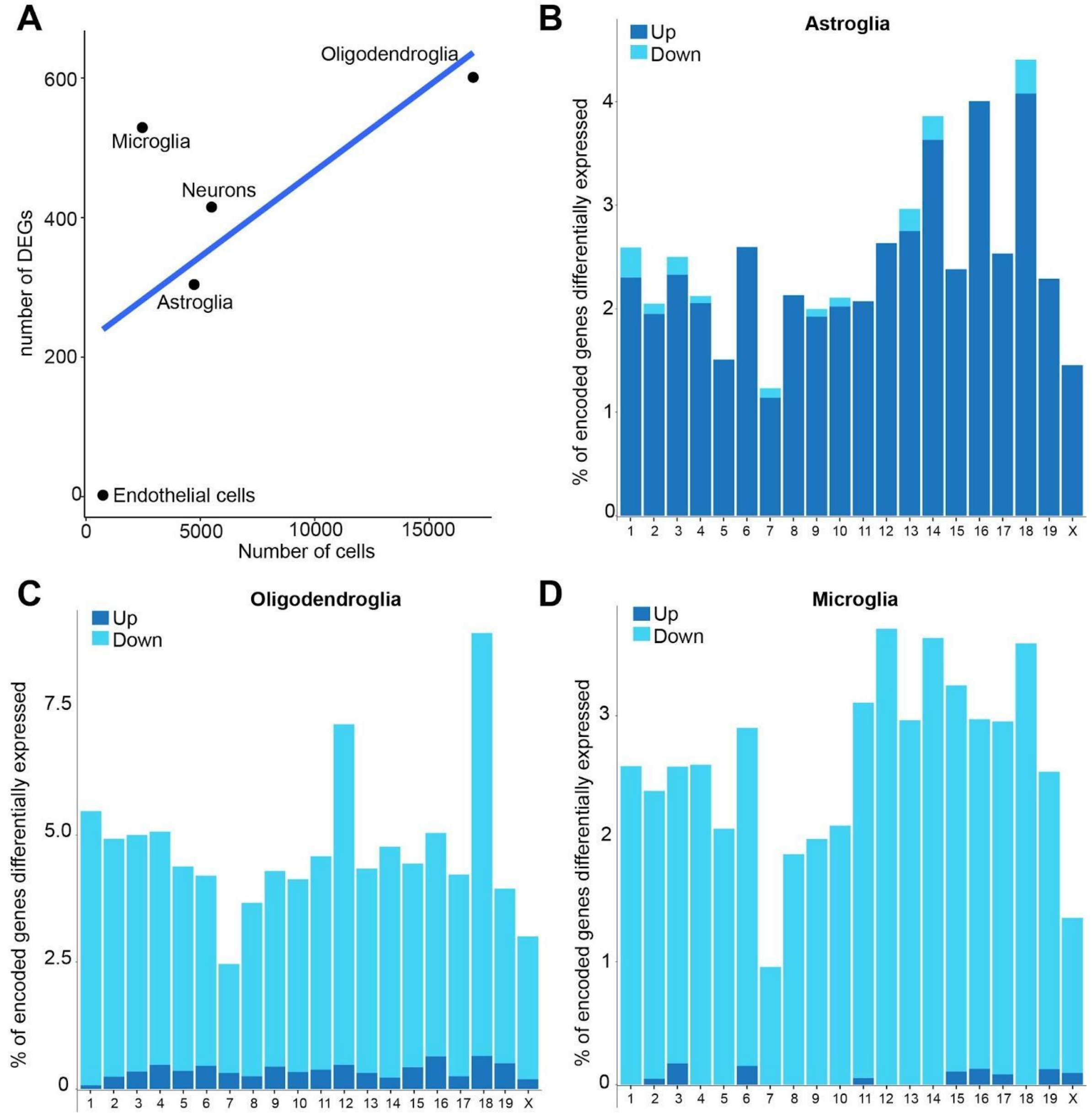

Supplementary Figure S3. (A) Correlation between the number of DEGs and the number of cells for the different cellular populations **(B)** Distribution of DEGs along the mouse chromosomes in the astroglia population. **(C)** Distribution of DEGs along the mouse chromosomes in the oligodendroglia population. **(D)** Distribution of DEGs along the mouse chromosomes in the microglia population.

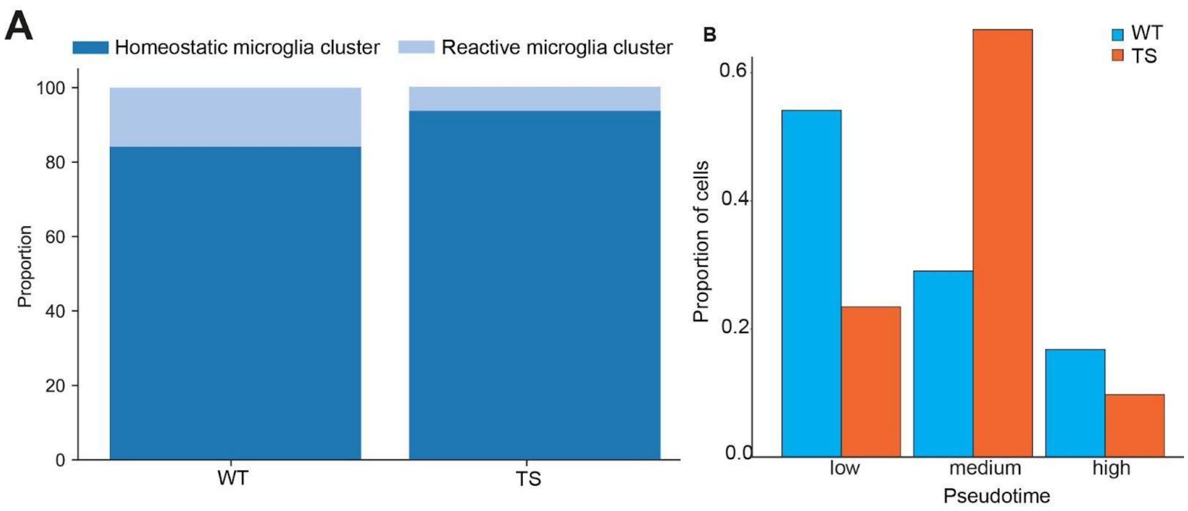

Supplementary Figure S4. (A) Proportion of cells that are within homeostatic microglial cluster (MG_0) and reactive microglial cluster (MG_1) in WT and in Ts65Dn mice. **(B)** Proportion of cells that are within the different pseudotime scores (low, medium and high) both in WT and in Ts65Dn mice.

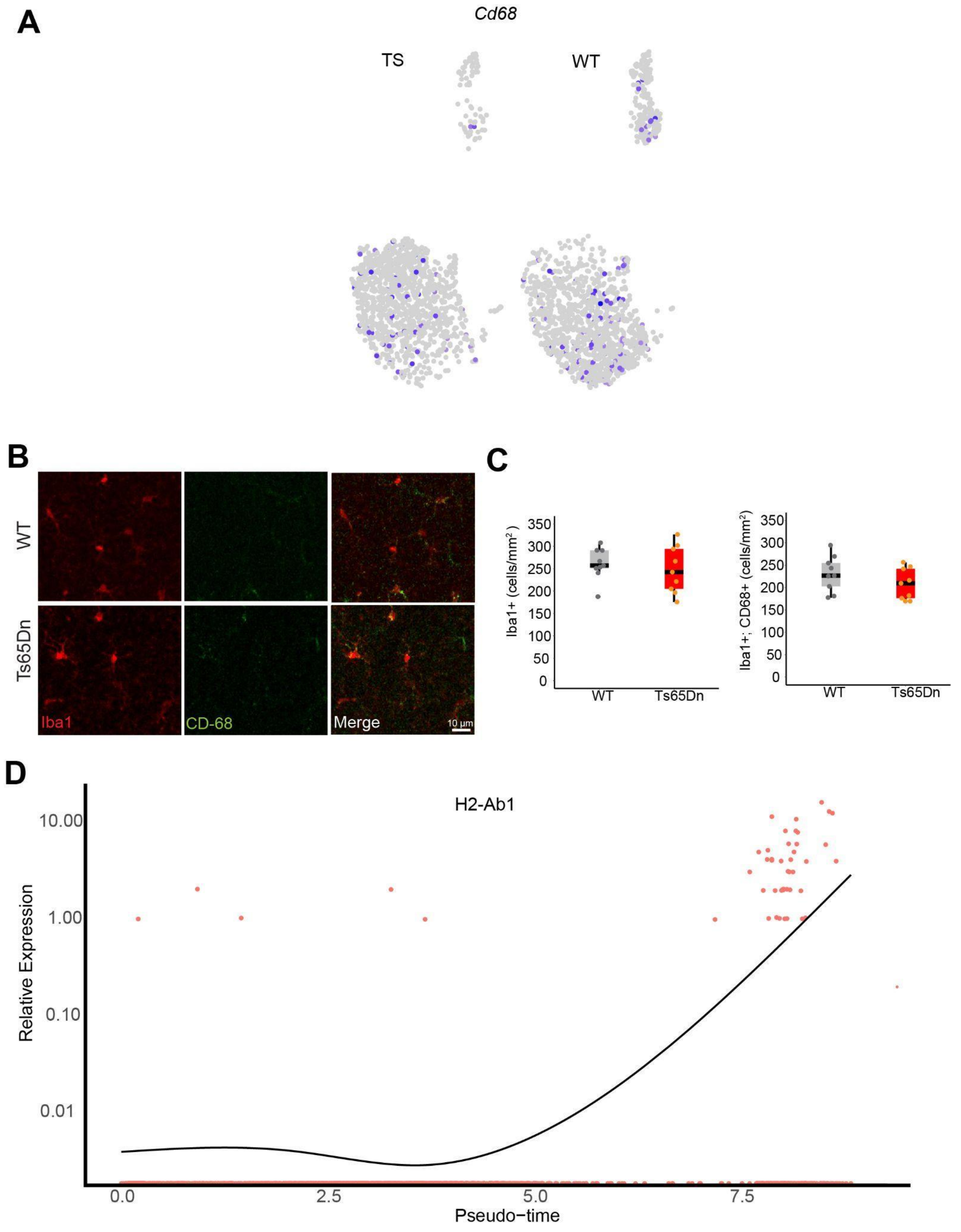

Supplementary Figure S5. (A) Representation of the two-dimensional embedding of TS and euploid microglial cells by diffusion map representing the expression of Cd68 marker. **(B)** Representative image of WT (above) and Ts65Dn (below) showing the Iba1+ cells (red), CD-68 signal (green) and merge image. **(C)** Quantification of Iba1+ cells (above) and Iba1+; CD-68+ cells (below) in CA1 stratum radiatum of WT and Ts65Dn mice (WT = 9 sections from 3 mice, Ts65Dn = 9 sections from 3 mice). Two-tailed T test. On the boxplots, the horizontal line indicates the median, the box indicates the first to third quartile of expression and whiskers indicate 1.5 × the interquartile range. **(D)** Relative expression of H2-Ab1 across the different microglial states in the pseudotime.

Supplementary Tables

Supplementary Table S1. Differentially expressed genes in individual cell types. Statistically significant DEGs are depicted in the Gene column and grouped by cell type (Astrocytes, Microglia, Oligodendrocytes). A negative avg_log2FC value indicates a downregulation while a positive value indicates upregulation compared to euploid controls. pct.1 is the percentage of single cells in which a gene is expressed for the specific cell type and pct.2 is the percentage expression in all other single cells.

https://crgcnag-my.sharepoint.com/:f:/g/personal/csierra_crg_es/Esrwr5mSzk5IvvC2lopxRIMBOtUymZOwxpc1V2HjzVhKdg?e=cJbHIU

Supplementary Table S2. Cluster-specific marker genes for the different cellular subpopulations. Columns A-I give statistics on cell type marker genes. WT and TS_avg_log2FC is the log fold change in the expression levels of a gene between the specific cell type and all the other cell types for WT and TS groups, respectively. pct.1 is the percentage of single cells in which a gene is expressed for the specific cell type and pct.2 is the percentage expression in all other single cells. Minimum_p_val is the combined p value between WT and TS groups. Columns K-O give the marker gene lists for the hippocampal cell types

https://crgcnag-my.sharepoint.com/:f:/g/personal/csierra_crg_es/Esrwr5mSzk5IvvC2lopxRIMBOtUymZOwxpc1V2HjzVhKdg?e=cJbHIU

Supplementary Table S3. Identity markers of the homeostatic and reactive microglial clusters. Columns A and B indicate the specific marker genes of the homeostatic (cluster_0) and reactive (cluster_1) clusters, respectively.

https://crgcnag-my.sharepoint.com/:f:/g/personal/csierra_crg_es/Esrwr5mSzk5IvvC2lopxRIMBOtUymZOwxpc1V2HjzVhKdg?e=cJbHIU
